# Benefits of Combined GPS/GLONASS with Low-Cost MEMS IMUs for Vehicular Urban Navigation

**DOI:** 10.3390/s120405134

**Published:** 2012-04-19

**Authors:** Antonio Angrisano, Mark Petovello, Giovanni Pugliano

**Affiliations:** 1 Department of Applied Sciences, Parthenope University of Naples, Centro Direzionale di Napoli, Isola C4, 80143 Napoli, Italy; 2 Department of Geomatics Engineering, Schulich School of Engineering, University of Calgary, 2500 University Drive NW, Calgary, AB T2N 1N4, Canada; E-Mail: mark.petovello@ucalgary.ca; 3 Department of Technology, Parthenope University of Naples, Centro Direzionale di Napoli, Isola C4, 80143 Napoli, Italy; E-Mail: giovanni.pugliano@uniparthenope.it

**Keywords:** GPS, GLONASS, Kalman filter, loosely coupled, tightly coupled, pseudo-observations

## Abstract

The integration of Global Navigation Satellite Systems (GNSS) with Inertial Navigation Systems (INS) has been very actively researched for many years due to the complementary nature of the two systems. In particular, during the last few years the integration with micro-electromechanical system (MEMS) inertial measurement units (IMUs) has been investigated. In fact, recent advances in MEMS technology have made possible the development of a new generation of low cost inertial sensors characterized by small size and light weight, which represents an attractive option for mass-market applications such as vehicular and pedestrian navigation. However, whereas there has been much interest in the integration of GPS with a MEMS-based INS, few research studies have been conducted on expanding this application to the revitalized GLONASS system. This paper looks at the benefits of adding GLONASS to existing GPS/INS(MEMS) systems using loose and tight integration strategies. The relative benefits of various constraints are also assessed. Results show that when satellite visibility is poor (approximately 50% solution availability) the benefits of GLONASS are only seen with tight integration algorithms. For more benign environments, a loosely coupled GPS/GLONASS/INS system offers performance comparable to that of a tightly coupled GPS/INS system, but with reduced complexity and development time.

## 1. Introduction

As is well known, urban environments are critical locations for Global Navigation Satellite Systems (GNSS). In such environments, buildings block many of the signals, thus reducing satellite availability and weakening observation geometry, with the extreme case being solution unavailability. Buildings can also reflect signals, causing multipath phenomena which introducs the greatest measurement errors in these areas. Past research on this problem can be broadly classified as focusing on: (a) increasing the number of satellites, usually by including additional GNSS to an existing system, or (b) integrating GNSS with external sensors, most commonly an inertial navigation system (INS).

With a few exceptions, the U.S. Global Positioning System (GPS) has been the primary GNSS since its inception many years ago. The Russian GLONASS system saw some use in the mid to late 1990s before suffering setbacks that plagued the system until the last few years. Nevertheless, the benefits of integrating GLONASS with GPS have been fairly well documented and improvements in measurement and solution availability, accuracy and reliability of positioning, and ambiguity resolution have been shown [1–3]. With the recent re-emergence of the GLONASS system, it is once again being considered for used in many systems (ibid.).

Integrating GNSS with INS has been very actively researched for many years due to the complementary nature of the two systems. In challenging GNSS environments like urban canyons and under foliage, the goal of the INS is to provide a navigation solution during GPS outages. Moreover the integration of GNSS with an inertial navigation system can deliver more robust and reliable systems than either of the individual systems alone [4]. The performance of the INS, however, is largely dependent on the quality (and thus cost) of the inertial sensors used, with higher quality sensors producing the best results.

To this end, use of high-end INS is generally limited to high accuracy applications only, owing to their price and size [5,6]. That said, recent advances in Micro Electro-Mechanical Systems (MEMS) technology have made possible the development of a new generation of low cost inertial sensors characterized by small size and light weight, which represent an attractive option for commercial applications such as pedestrian and vehicular navigation. MEMS-based INS are characterized by low performance too, especially in the absence of GNSS data, so their use as part of an integrated navigation system is currently under investigation. In the last few years several researchers have investigated the integration of GNSS systems with MEMS-based INS [5–12]. It is worth noting however, that all of these studies have focused only on GPS for updating the INS.

The integration of combined GPS/GLONASS with INS was already tested several years ago (e.g., by Lechner *et al*. [13]) or more recently, for instance by Rinnan *et al*. [14], but with high-end IMU devices), it follows the idea to test in this work the GPS/GLONASS performance with low-cost inertial sensors.

For vehicular navigation in particular, constraints on the vehicle's velocity—known as non-holonomic constraints—can be applied to further improve INS performance [6,15–19]. In addition, GNSS-derived yaw (azimuth) can also be used to improve performance [11]. Finally, by assuming the vehicle is running on an approximately level road, a height constraint can also be enforced.

The primary objective of this paper is to expand the previous work in order to investigate the benefits, if any, of adding GLONASS to GPS/INS (MEMS) systems, specifically for vehicular navigation applications. As part of this, the paper assesses the performance of loose and tight integration algorithms (*i.e*., position-level and measurement-level integration, respectively). In particular, since loose coupling cannot provide updates during periods of insufficient satellite visibility [5], thus causing decreased performance relative to the tight integration case, the role of adding GLONASS data to the different architectures is considered (an interesting application of GNSS/INS integration with loosely coupled strategy is performed in [20]). In a similar manner, the role of non-holonomic constraints, as well as aiding information from GNSS-derived heading and from height constraints must also be re-evaluated given the improvement in GNSS measurements availability.

With this in mind, the main contributions of the paper are as follows: first, by adding GLONASS to existing GPS/INS systems, the role of the improved satellite availability on system accuracy is assessed in an urban environment. Second, the relative performance of loose and tight integration algorithms is assessed with and without GLONASS. In so doing, it is shown that in some cases the loose integration approach with GLONASS can yield similar performance to the GPS-only case with a tight integration. This information is useful to system designers since loose integration algorithms are generally easier to implement than their tight integration counterparts. Third, the benefit of non-holonomic constraints, GNSS-derived yaw aiding, and height constraints are also assessed in the presence of GLONASS to assess their benefits relative to the GPS-only case. Finally, it is worth noting that although the focus is on the role of GLONASS for operational reasons, the results presented should also apply to other upcoming GNSS such as Galileo and/or Compass.

The rest of the paper is organized as follows: first, the relevant theories in the context of this paper are briefly reviewed with focus given to the different integration algorithms (loose *versus* tight coupling) and the available aiding information (GNSS-derived heading and non-holonomic constraints). Second, the tests used to assess the performance of the various integration strategies are described. Results are then presented and analyzed, followed by a summary of the main conclusions.

## 2. Systems Overview

This section gives a brief overview of GPS and GLONASS as well as the inertial sensors used in the final integrated system.

### 2.1. GPS/GLONASS

GPS and GLONASS are the main GNSS systems in use today and they are similar in many respects, but with some essential differences. Both systems are able to provide various number of air, marine, and any other type of users with all-weather three-dimensional positioning, velocity and timing, anywhere in the World or near-Earth space. Both navigation systems are based on the concept of “one-way ranging”, in which the unknown user position is obtained measuring the time of flight of signals broadcasted by satellites at known positions and epochs [21].

The main difference between the two systems is that GPS and GLONASS operate with different time references and with different coordinate frames [22,23]. Specifically, GPS time is related with UTC (USNO), Coordinated Universal Time (UTC) as maintained at the United States Naval Observatory. In contrast, GLONASS time is related to UTC (SU), UTC as maintained by Russia. The offset between the two time references can be calibrated, but this information is not yet included in the navigation messages broadcasted by the satellites. This causes an increase in the number of unknowns to be estimated from 4 to 5; three coordinates of user position and the biases of the receiver clock relative to the two system time scales (one bias can be replaced by the inter-systems time offset). This problem will eventually be overcome with the new generation of GLONASS satellites (*i.e*., GLONASS-M) that are planned to broadcast the offset between the two time scales. In addition, the GPS and GLONASS datum difference does not require an additional state because WGS84 and PZ90 are known and fixed, and they are linked by a well-defined mathematical transformation (further details are given in [24]). Other differences are related to the signal nature, namely different signal bandwidths and multiple access schemes, which are not relevant to this paper.

### 2.2. Low-Cost Inertial Sensors

The great advances in MEMS have made possible the development of a new generation of low cost inertial sensors. MEMS inertial measurement units (IMU), that is, the actual sensor assembly, are characterized by small size, light weight, low cost and low power consumption with respect to higher-end inertial sensors. These features make MEMS sensors an attractive option for applications such as vehicular navigation. However, MEMS sensors are also characterized by poorer performance, so they cannot be used in autonomous mode for extended periods although they are well suited for integrated navigation systems (usually coupled with GPS systems) where external measurements can limit their error growth. An IMU usually consists of a triad of accelerometers and gyros, whose measurement equation can be expressed as:

(1) $$\begin{array}{c} f=\tilde{f}+b_{a}+f\cdot S_{a}+\eta_{a}\\ \omega =\tilde{\omega }+b_{g}+\omega \cdot S_{g}+\eta_{g} \end{array}$$

with *f* and *f̃* being the actual and measured specific force,

*ω* and *ω̃* actual and measured angular rate,
*b_a_* and *b_g_* sensor biases of the accelerometer and gyro respectively,
*S_a_* and *S_g_* sensor scale factors of the accelerometer and gyro respectively,
*η_a_* and *η_g_* sensor noises of the accelerometer and gyro respectively.

More detailed measurement equations can be found in [25], including additional terms such as non-linear scale factors and cross-axis coupling factors. These are not considered here as they are impractical to estimate given the amount and quality of GNSS data used in this research.

The sensor bias is defined as the average of the output, obtained during a specific period with fixed operational conditions when the input is null. The bias generally consists of two parts: a deterministic part called bias offset or turn-on bias and a stochastic part called bias drift or in-run bias. The turn-on bias is essentially the offset in the measurement and is constant over a single mission; it has deterministic nature and so can be determined by calibration procedure (or it can be also modeled statistically as a random constant process). The bias drift is the change in the sensor with time; the bias drift has random nature and so must be modeled as a stochastic process. The scale factor error is the ratio between the change in the output signal of the sensor to the change in the measured physical quantity. In ideal conditions the scale factor should be unity. This error has a deterministic nature but generally is modeled as a random process. The inertial sensor errors can be expressed in terms of angular random walk (ARW) and velocity random walk (VRW). The ARW parameter describes the average deviation or error that will occur from integrating the noise on gyro output signal. Similarly the VRW parameter definition is based on the same concept for the accelerometers.

Typical MEMS sensor performance is summarized in the Table 1, where navigation and tactical grade IMU performance is also listed for comparison. Since gyro biases degrade position as a function of time cubed [26], it is obvious from the table that MEMS-based sensors will produce very poor navigation results in a short time unless integrated with other systems (typically GNSS) to bound the errors. From Table 1 we can see that the turn-on bias of MEMS gyro is about 5,400 deg/h, while it is considerably smaller in the navigation and tactical grade sensors. Also the in-run bias can be 1,040 deg/h in MEMS sensors, while is about 1 deg/h in a tactical grade gyro. These parameters provide a good assessment of MEMS performance with respect to higher grade sensors.

## 3. Integrated Navigation

Before looking at the GNSS/INS integration algorithms, GNSS-only processing is briefly reviewed. To this end, in this work, the GNSS measurements are processed in single point mode, so no differential corrections are applied and the deployment of a reference station is unnecessary. Only pseudorange and Doppler (phase rate) observables are used.

To account for the fact that satellite measurements at low elevation angles are generally noisier [28], the measurements are weighted the sine of the satellite elevation angle, as proposed in [19,27]. To consider also the different accuracy related to the pseudorange and Doppler observables, the weight (reciprocal of variance) associated to the generic measurement is expressed by:

(2) $$w_{ii}=\sin\left(el\right)/\sigma_{m}^{2}$$

where $\sigma_{m}^{2}$ is either the pseudorange variance $\sigma_{PR}^{2}$ or the pseudorange rate variance $\sigma_{\text{PRdot}}^{2}$.

The GNSS solution is obtained using the WLS (Weighted Least Squares) method, whose equation is:

(3) $$\underline{\Delta x}={\left(H^{T}WH\right)}^{-1}H^{T}W\underline{\Delta \rho }$$

with Δ*ρ* being the measurement misclosure vector,

*H* being the geometry (design) matrix,
Δ*x* being the unknown vector of corrections to the current state estimates, and
*W* being the diagonal weighting matrix whose elements *w_ii_* are from Equation (2).

The states are position, velocity and clock errors. If a single GNSS system is considered (e.g., GPS or GLONASS only) the clock error is modeled by two states: a bias and a drift. If two GNSS are combined (e.g., GPS/GLONASS case) a further state, representing the inter-systems time offset, must be included.

### 3.1. GNSS/INS Integration

GNSS/INS integration is very common because the systems are complementary in many aspects. In particular, INS is more accurate in the short term, it can supply data with very high rate and it can also provide attitude information. On the other hand, GNSS is more accurate in the long term and the error is effectively time invariant. The following sections describe the two most common approaches to integrating GNSS and inertial data, namely loose and tight coupling.

#### 3.1.1. Loosely Coupled Approach

**Figure 1.**
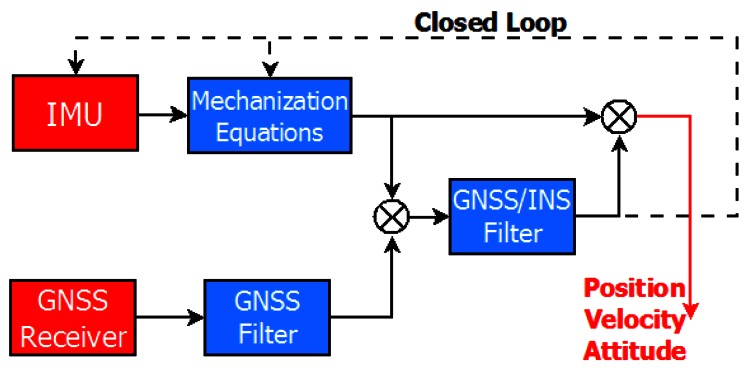
Loosely coupled scheme.

The loose coupling (LC) strategy is also referred to as “decentralized” and includes a Kalman Filter (KF) to combine INS and GNSS parameters. Another KF or a LS estimator is used to compute the GNSS navigation solution. The LC scheme is showed in Figure 1. Although the LC approach is relatively simple to implement, the main drawback of the LC approach is that if there are insufficient satellites to compute a standalone GNSS solution, the inertial system is not updated. This ultimately results in higher positioning errors [27] relative to the tight coupling approach (details to follow).

To compute the GNSS fix, a LS estimator is preferred herein to simplify a direct LC/TC comparison. Specifically, by using least-squares for LC, the results will be the same as in the TC case so long as enough satellites are available to compute a solution.

The inertial solution is obtained by applying the mechanization equations for a strapdown configuration to the accelerations and angular rates from the IMU. For this work, the INS mechanization is implemented in the local East-North-Up (ENU) frame. Details of the mechanization equations are widely available in the literature (e.g., [26]) and are thus excluded here.

The difference between the INS and weighted least-squares (WLS) GNSS position and velocity are used as input measurements to the KF. The WLS covariance matrix is used as measurements covariance matrix *R* for the KF:

(4) $$R=\mathrm{cov}\left(\underline{\Delta x}\right)={\left(H^{T}WH\right)}^{-1}$$

Implicit in the above is that only the sub-matrices corresponding to the states being used in the update are extracted from the WLS covariance matrix (details follow shortly).

The state vector of the combined GNSS/INS KF in LC architecture is:

(5) $$\underline{\delta x}={\left[\begin{array}{ccc} \begin{array}{ccccc} {\underline{\delta P}}^{n} & {\underline{\delta v}}^{n} & {\underline{ɛ}}^{n} & {\underline{\delta b}}_{a} & {\underline{\delta b}}_{g} \end{array} & {\underline{\delta S}}_{a} & {\underline{\delta S}}_{g} \end{array}\right]}^{T}$$

with *δP^n^* position error vector, *δv^n^* velocity error vector, *ε^n^* attitude error vector, *δb_a_* accelerometer bias error vector, *δb_g_* gyro bias error vector, *δS_a_* accelerometer scale factor vector and *δS_g_* gyro scale factor. The GNSS receiver clock errors are not included because they are easily separated from the other states after the WLS solution.

The INS error model (for position, velocity and attitude) is typical of what is widely available in the literature (e.g., [26]). The bias error vectors *δb_a_*, *δb_g_* are modeled as 1st order Gauss-Markov processes and include the sum of the in-run and turn-on biases [19]. The scale factor vectors *δS_a_*, *δS_g_* are also modeled as 1st order Gauss-Markov processes (ibid.). The MEMS-based IMU used in this work is the Crista IMU, whose random noise spectral density and Gauss-Markov (GM) parameters are shown in Table 2.

#### 3.1.2. Tightly Coupled Approach

**Figure 2.**
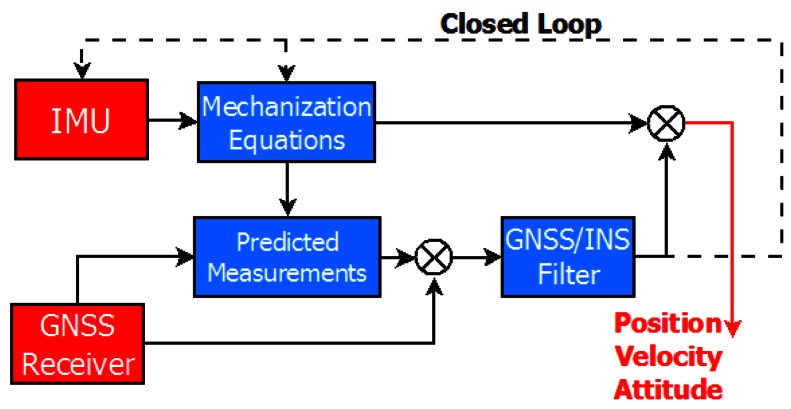
Tightly coupled scheme.

The tight coupling (TC) strategy is also referred to as “centralized”, because there is only one central KF processing both the GNSS observations and INS data. The TC scheme is showed in Figure 2. Although this approach is more complicated, it generally shows better performance because updates can be performed regardless of how many satellites are being tracked.

In contrast to the LC case, the difference between the measured and INS-predicted pseudorange and Doppler observables is used as input measurements to KF. The associated measurement covariance matrix is defined taking into account the inherent accuracies of GNSS measurements and the elevation-dependent accuracy as in the LC case (see Equation (2)).

The TC KF state vector has the same 21 states as for the LC case (see Equation (5)), but is also augmented with the GPS receiver clock bias and drift. If GLONASS measurements are included, the GPS-GLONASS inter-systems time offset must also be considered and in this work it is modeled as a random constant.

Both loose and tight strategies are herein implemented in closed loop configuration meaning the navigation, bias and scale factor error states output from the KF are used to correct INS inputs. The closed loop configuration is necessary when low performance INS is used to reduce the inertial error growth [6], which in turn, satisfies the small angle assumptions used to derive the INS error equations.

### 3.2. Aiding Methods

This section briefly looks at the details of using GNSS-derived heading, as well as pseudo-observations of vehicle velocity and height to help improve the overall solution.

#### 3.2.1. GNSS-Derived Heading Aiding

A GNSS-derived aiding can be used to improve the heading estimation. To incorporate heading measurements into the measurement model of the GNSS/INS filter, an equation relating the heading errors with the system error states is required [5]. Such an error equation is derived starting from the rotation matrix from East-North-Up (ENU) to body frame $R_{n}^{b}$:

$$R_{n}^{b}=\left(\begin{array}{ccc} \cos\phi \cos\psi +\sin\phi \sin\theta \sin\psi & -\cos\phi \sin\psi +\sin\phi \sin\theta \cos\psi & -\sin\phi \cos\theta \\ \cos\theta \sin\psi & \cos\theta \cos\psi & \sin\theta \\ \sin\phi \cos\psi -\cos\phi \sin\theta \sin\psi & -\sin\phi \sin\psi -\cos\phi \sin\theta \cos\psi & \cos\phi \cos\theta \end{array}\right)$$

where, *Ø θ*, *ψ* are respectively roll, pitch and azimuth angles.

The expression of the azimuth as function of the elements of the matrix $R_{n}^{b}$and of its inverse $R_{b}^{n}$is:

(6) $$\hat{\Psi }=\arctan\left(\frac{{\hat{R}}_{n}^{b}(2,1)}{{\hat{R}}_{n}^{b}(2,2)}\right)=\arctan\left(\frac{{\hat{R}}_{b}^{n}(1,2)}{{\hat{R}}_{b}^{n}(2,2)}\right)$$

where the hats on the top remark that the quantities are estimated.

The relationship between estimated and actual rotation matrix from body to ENU frame is

$${\hat{R}}_{b}^{n}=\left(I-E^{n}\right)R_{b}^{n}$$

with *E^n^* = [*ε_E_ ε_N_ ε_U_*]*^T^* skew-symmetric matrix of the vector of the attitude errors in the ENU frame.

With this in mind the Equation (6) becomes:

$$\hat{\psi }=\arctan\left(\frac{{\hat{R}}_{b}^{n}\left(1,2\right)}{{\hat{R}}_{b}^{n}\left(2,2\right)}\right)=\arctan\left(\frac{R_{b}^{n}\left(1,2\right)+{ɛ}_{U}R_{b}^{n}\left(2,2\right)-{ɛ}_{N}R_{b}^{n}\left(3,2\right)}{-{ɛ}_{U}R_{b}^{n}\left(1,2\right)+R_{b}^{n}\left(2,2\right)+{ɛ}_{E}R_{b}^{n}\left(3,2\right)}\right)$$

Computing the differential of the previous equation, the heading error equation is obtained as:

(7) $$\delta \psi =\frac{\partial \psi }{\partial {ɛ}_{E}}\delta {ɛ}_{E}+\frac{\partial \psi }{\partial {ɛ}_{N}}\delta {ɛ}_{N}+\frac{\partial \psi }{\partial {ɛ}_{U}}\delta {ɛ}_{U}$$

with:

$$\begin{array}{c} \frac{\partial \hat{\Psi }}{\partial {ɛ}_{E}}=\;\frac{{\hat{R}}_{b}^{n}\left(1,2\right)R_{b}^{n}(3,2)}{{\left[{\hat{R}}_{b}^{n}\left(2,2\right)\right]}^{2}+{\left[{\hat{R}}_{b}^{n}\left(1,2\right)\right]}^{2}}\approx \frac{{\hat{R}}_{b}^{n}\left(1,2\right){\hat{R}}_{b}^{n}(3,2)}{{\left[{\hat{R}}_{b}^{n}\left(2,2\right)\right]}^{2}+{\left[{\hat{R}}_{b}^{n}\left(1,2\right)\right]}^{2}}\\ \frac{\partial \hat{\Psi }}{\partial {ɛ}_{N}}=\;\frac{{\hat{R}}_{b}^{n}\left(2,2\right)R_{b}^{n}(3,2)}{{\left[{\hat{R}}_{b}^{n}\left(2,2\right)\right]}^{2}+{\left[{\hat{R}}_{b}^{n}\left(1,2\right)\right]}^{2}}\approx \frac{{\hat{R}}_{b}^{n}\left(2,2\right){\hat{R}}_{b}^{n}(3,2)}{{\left[{\hat{R}}_{b}^{n}\left(2,2\right)\right]}^{2}+{\left[{\hat{R}}_{b}^{n}\left(1,2\right)\right]}^{2}}\\ \frac{\partial \hat{\Psi }}{\partial {ɛ}_{U}}=\;\frac{{\hat{R}}_{b}^{n}\left(1,2\right)R_{b}^{n}\left(1,2\right)+{\hat{R}}_{b}^{n}\left(2,2\right)R_{b}^{n}\left(2,2\right)}{{\left[{\hat{R}}_{b}^{n}\left(2,2\right)\right]}^{2}+{\left[{\hat{R}}_{b}^{n}\left(1,2\right)\right]}^{2}}\approx 1 \end{array}$$

In Equation (7) the measurement is:

(8) $$\delta \psi =\left[\psi_{\text{INS}}-\psi_{\text{GNSS}}\right]=\left[\psi_{\text{INS}}-\arctan\left(\frac{V_{E}}{V_{N}}\right)\right]$$

with *ψ_INS_* being the azimuth estimated in the GNSS/INS filter, *ψ_GNSS_* being the GNSS-derived azimuth depending on East and North velocity components and whose accuracy is expressed as:

(9) $$\sigma_{\psi }^{2}=\frac{\sigma_{V_{\text{GNSS}}}^{2}}{V_{H_{\text{GNSS}}}^{2}}$$

where *V_HGNSS_* is the horizontal speed (estimated by GNSS) and $\sigma_{V_{\text{HGNSS}}}^{2}$ is the associated variance.

The external heading equation can be embedded in the measurement model of the GNSS/INS KF and is used when the horizontal speed of the vehicle is sufficiently high. For this work, this measurement is only used when the vehicle's speed exceeds 5 m/s, as this gave reasonable results. Other thresholds were not investigated, and this is left as future work.

#### 3.2.2. Non-Holonomic Constraints and Pseudo-Measurements of Height

In vehicular navigation one possible approach to mitigate INS error accumulation is to apply constraints based on the motion of the vehicle. In other words, it is possible to generate constraint equations reflecting the behavior of the vehicle during navigation [15–17].

In the context of this paper, it is assumed that the vehicle does not slip sideways or jump/bounce such that the motion is essentially in the vehicle's longitudinal direction. The above assumptions constitute the so called non-holonomic constraints and are described mathematically as:

(10) $$\begin{array}{c} v_{x}^{b}=\eta_{x}\\ v_{z}^{b}=\eta_{z} \end{array}$$

where $v_{x}^{b}$ and $v_{z}^{b}$ are the velocity components in body's (vehicle's) lateral and upward directions respectively, and *η_x_* and *η_z_* are the (fictitious) measurement noises denoting the possible discrepancies in the above stated assumptions. The latter are needed since there may be some small motions in the lateral and vertical directions.

Equation (10) can be used during GNSS outages to aid the INS navigation using the following equation:

(11) $${\underline{\delta V}}^{b}=R_{n}^{b}{\underline{\delta V}}^{n}+R_{n}^{b}{\underline{V}}^{n}\times {\underline{ɛ}}^{n}$$

which expresses how velocity aiding in the body frame, *δv^b^*, can improve velocity and attitude estimation.

A second constraint equation can be generated considering the fact that the height does not change significantly in land navigation over short time periods. Hence during GNSS outages the height can be considered constant and equal to a reference value *h_ref_*, computed just before the outage in good visibility condition [19]. Mathematically, this is written as:

(12) $$h\approx h_{\text{ref}}$$

Although this may seem equivalent to the second equation in Equation (10), there is a subtle difference. In particular, Equation (10) refers to the body frame, whereas the height pseudo-observation applies in the local level frame. Since it is possible that the body frame is inclined relative to the true vertical (e.g., if the car is tilted due to uneven weight distribution), these two constraints are not, strictly speaking, equivalent.

**Figure 3.**
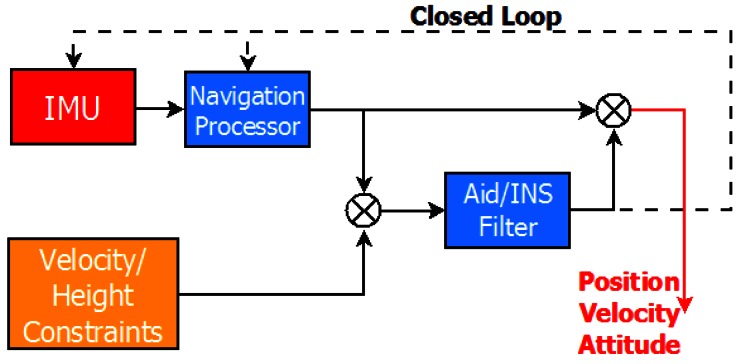
Velocity/height constraints aiding scheme.

Both the non-holonomic constraints and the pseudo-measurements of height can be used as measurement models of a complementary Kalman filter during GNSS outages as shown in Figure 3. As such, the non-holonomic and height constraints are herein respectively named velocity and height pseudo-measurements. Finally, although not shown in Figure 3, these can also be applied in the presence of GNSS updates.

## 4. Test Description and Equipment

A data collection experiment was carried out in a vehicle in downtown Calgary, Canada on 22 July 2010 in the afternoon around 2:00 pm local time. Downtown Calgary is a typical urban scenario, characterized by skyscrapers and so it is a difficult environment for satellite navigation because of blocking and multipath problems. The total duration of the test is about 23 min; the speed of the vehicle varied from 0–50 km/h with frequent stops due to the traffic lights and the total distance travelled is about 10 km.

The test equipment consists of a satellite receiver and a MEMS IMU (whose specifications are in Table 1) to perform the experiment and more accurate devices as reference. Specifically, the NovAtel Receiver ProPak V3—able to receive GPS and GLONASS satellite signals—and a Crista IMU from Cloud Cap Technology are used to test the different configurations. It is acknowledged that the use of a high quality receiver is inconsistent with the theme of a low-cost system. However, no low-cost GPS/GLONASS receiver was available for testing. Nevertheless, the differences obtained between using GPS-only and GPS and GLONASS together are expected to be largely receiver independent.

**Figure 4.**
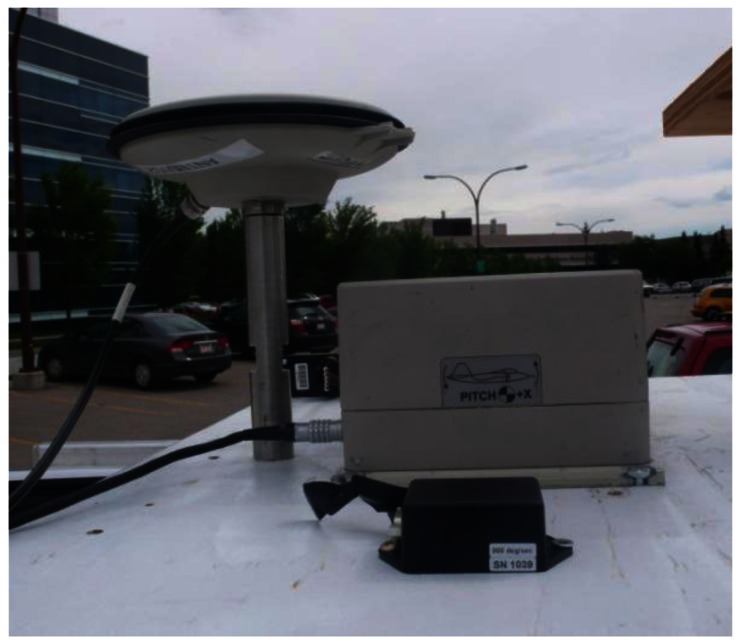
Equipment.

**Figure 5.**
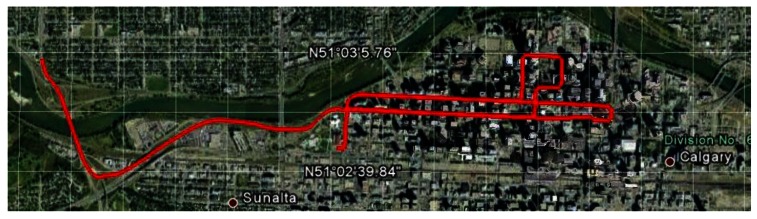
Test trajectory (from Google Earth).

The reference solution is obtained using the NovAtel SPAN (Synchronized Position Attitude Navigation), an integrated system consisting of the OEM4 NovAtel receiver and the HG1700 tactical grade IMU. The SPAN data are processed by NovAtel's Inertial Explorer software using phase and Doppler measurements in double difference mode. The baseline separation (relative to a base station located on the University of Calgary campus) varied between 6–7 km. The reference solution accuracy in these conditions is summarized in Table 3. All the equipment was placed on the roof of the car as showed in Figure 4 and the complete trajectory test is shown in Figure 5.

**Figure 6.**
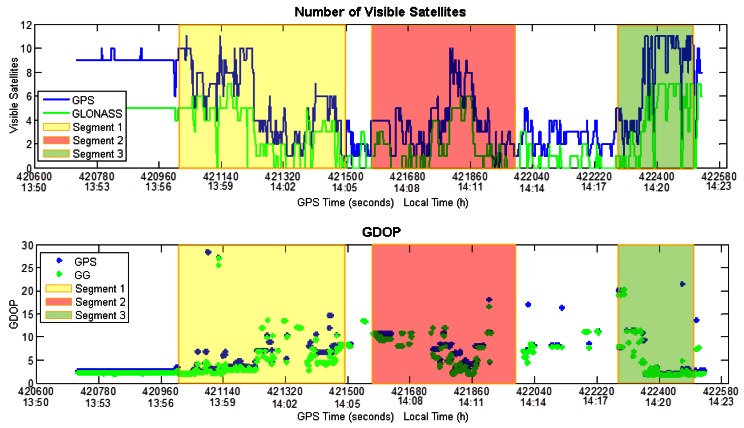
Visibility and GDOP during the test.

To facilitate the data analysis, three segments of the trajectory, representing different operational scenarios, are considered and examined separately. The visibility and GDOP behavior is shown in Figure 6, where also the segments are highlighted. The GDOP parameter is a geometry factor, representing the amplification of the standard deviation of the measurement errors onto the solution [21].

The main features of the GNSS coverage during all segments are summarized in Table 4, in terms of solution availability and maximum outage duration. This clearly shows the benefits of including GLONASS observations in this scenario.

**Figure 7.**
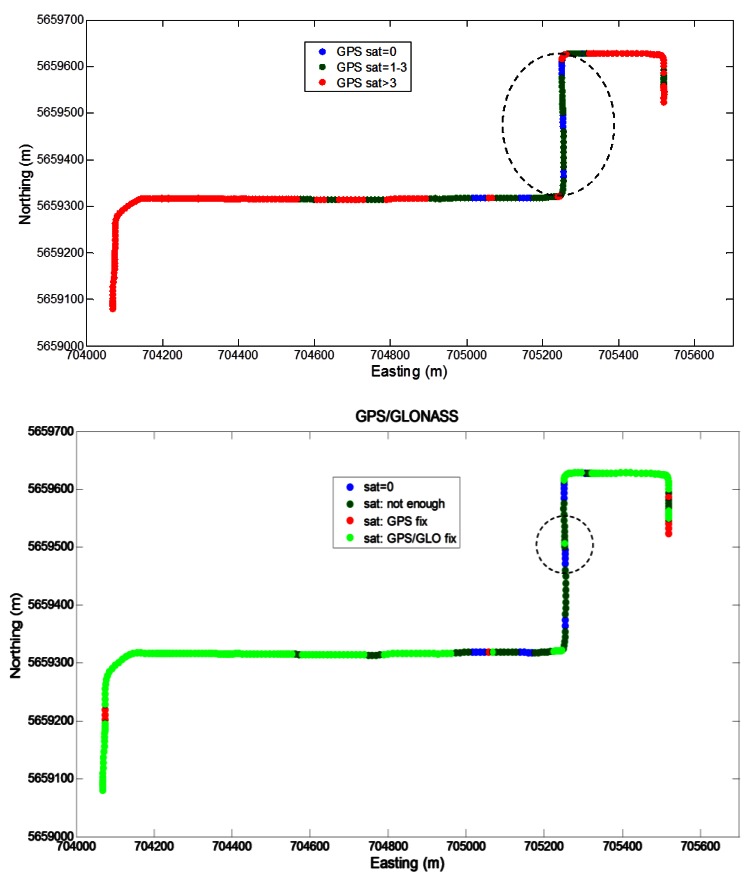
GNSS visibility on the Segment 1 trajectory.

The segment 1 started in a parking lot where the satellite visibility was good and the operational conditions can be considered semi-open sky (4–10 visible GPS satellites). The second part of the segment 1 was in a demanding urban canyon with poor satellite coverage (0–6 available GPS satellites). The satellite visibility in the GPS-only and GPS/GLONASS cases is shown in Figure 7. In the GPS-only case, frequent partial and total outages are evident, particularly towards the east end of the long east/west part of the trajectory and along the north/south part of the trajectory (located near the center of downtown Calgary). The longest GPS period during which a standalone GPS solution is unavailable (herein termed an “outage”) is about 60 s and is highlighted by a dotted circle in the top part of Figure 7. In the GPS/GLONASS case this period is still largely present, but is shortened by a combined GPS/GLONASS fix as shown in bottom part of Figure 7.

**Figure 8.**
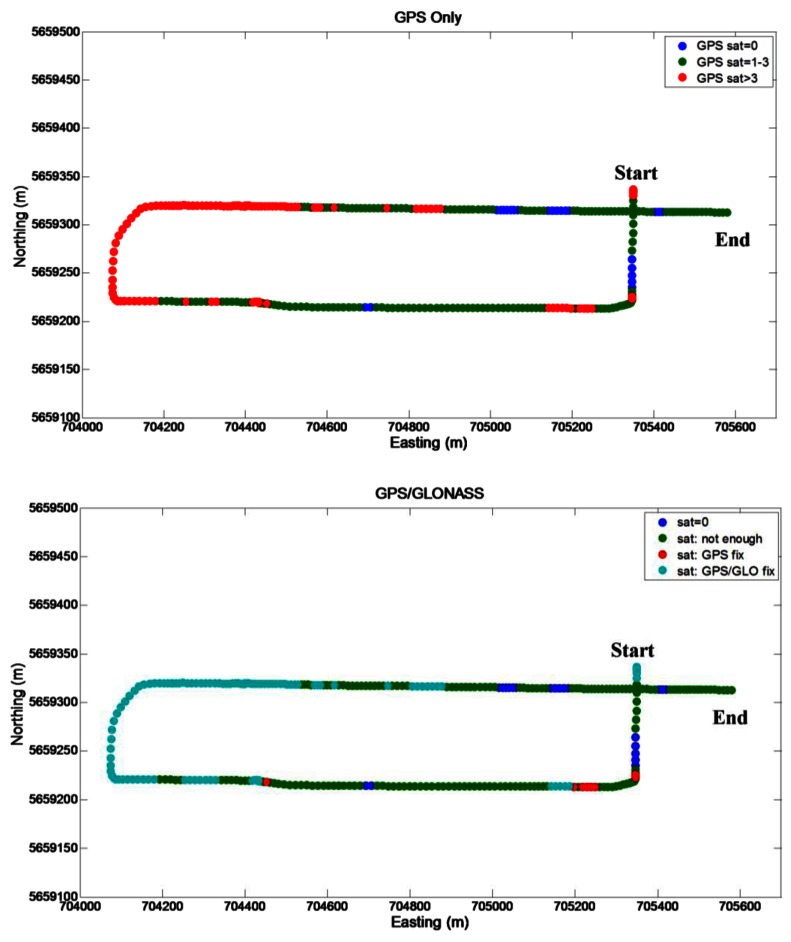
GNSS visibility on the Segment 2 trajectory.

Segment 2 represents a very difficult scenario. The number of visible GPS satellites on the trajectory is presented in Figure 8 (on the top), showing two long partial/total GPS outages (about 60 s) on the two east/west legs. These two parts of the trajectory are connected by a turn at western-most part of downtown with a sufficient visibility to continuously perform a GPS fix. The inclusion of GLONASS (Figure 8 on the bottom) does not improve significantly the satellite coverage during this segment as also shown in Table 4.

**Figure 9.**
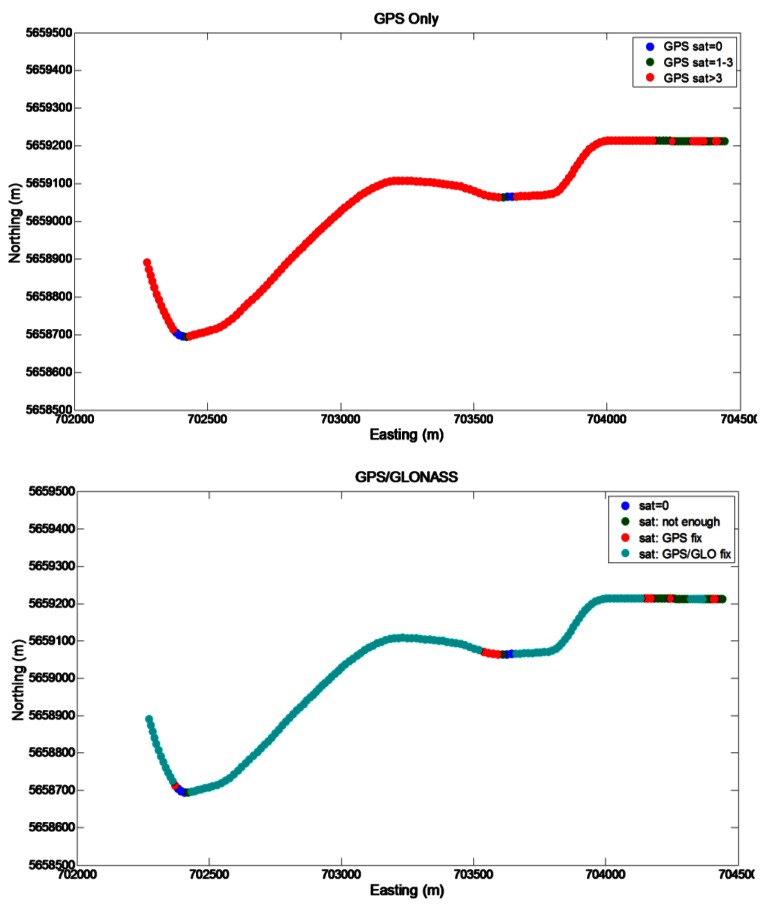
GNSS visibility on the Segment 3 trajectory.

Segment 3 represents a more benign scenario where the number of visible GPS satellites during the trajectory is presented in Figure 9 (on the top). After some short partial GPS outages (few seconds duration) at the beginning of the segment, the satellite visibility allows almost continuous GPS positioning. Only two short outages were encountered on the trajectory. Inclusion of GLONASS (Figure 9 on the bottom) further increases the number of available satellites, reducing the durations of the outages and increasing solution availability (Table 4).

## 5. Results and Analysis

As mentioned before, the primary objective of this work was to compare the performance of GPS and GPS/GLONASS integrated with low cost INS with a particular focus on assessing the benefits of including GLONASS. Both loose and tight integration strategies are tested to determine if the type of integration plays a significant role. The pseudo-measurements based on assumptions about the typical vehicular behavior are included in both integration architectures to assess the effectiveness in this context, as are GNSS-derived yaw observations.

To this purpose, several processing configurations are considered. The baseline configuration in the loose and tight integration cases are based on GPS alone and are respectively denoted as “LC GPS/INS” and “TC GPS/INS”. Similarly, the inclusion of GLONASS is denoted as “LC GG/INS” and “TC GG/INS”. The use of constraints are denoted by appending a Y (GNSS-derived yaw), V (velocity pseudo-measurement) and H (height pseudo-measurement). For example, LC GG/INS YVH represents the loosely couple GPS/GLONASS solution with all three constraints applied.

### 5.1. Loose Coupling Solutions

#### 5.1.1. Segment 1

**Figure 10.**
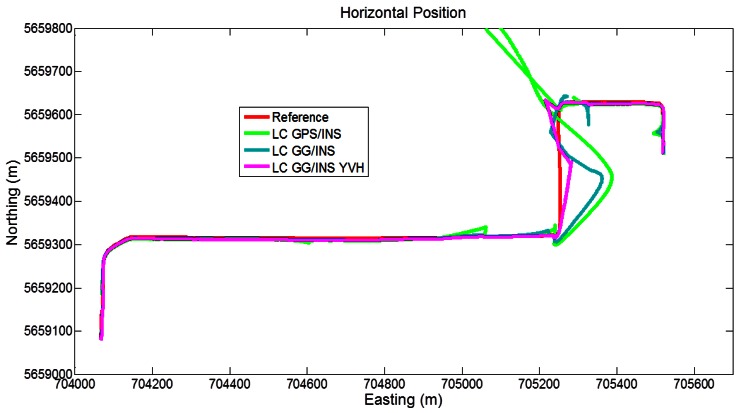
Trajectories obtained with the loose coupling approach (Segment 1).

In Figure 10 the trajectories obtained with the different LC approaches are shown. The GPS/INS solution shows large errors during the above-mentioned longest GPS outage, with maximum errors reaching about 400 m. Adding GLONASS to this solution shows evident improvements relative to the baseline case. In fact the isolated GPS/GLONASS fix (circled in right part of Figure 7) allows the trajectory to stay relatively near the reference between the second and the third turns, whereas without GLONASS much larger errors occur. In addition, the LC GG/INS configuration provides better performance than the GPS-only solution during the east-west stretch (around easting values of 705,000), where LC GPS/INS error tends to grow owing to short GPS only outage (15–20 s duration). Finally, the trajectory obtained with GPS/GLONASS/INS augmented with the GNSS-derived yaw aiding as well as velocity and height pseudo-measurements shows remarkable improvements relative to the other configuration with only small disagreements with the reference trajectory.

The contribution of various combinations of constraints to the position solution is summarized in Table 5. As can be seen, the yaw (GNSS-derived heading) provides little overall benefit. In contrast, the velocity provides a large benefit. The height constraint does not improve the RMS accuracy, but does reduce the maximum position error by about 28%.

#### 5.1.2. Segment 2

**Figure 11.**
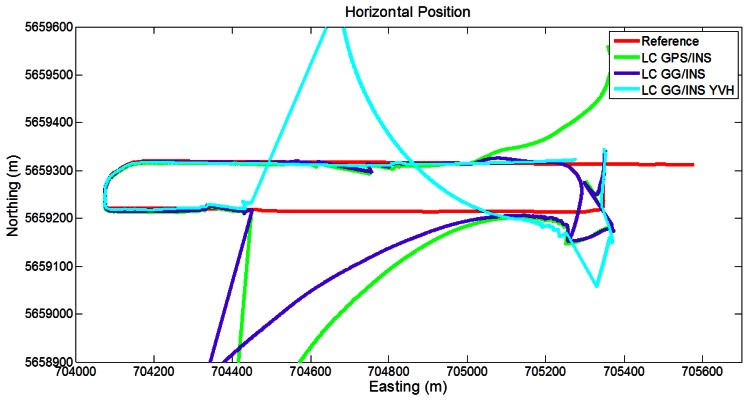
Trajectories obtained with the loose coupling approach (Segment 2).

Figure 11 shows the trajectories obtained with the different LC approaches. The GPS/INS solution presents large errors during the GPS outages. The inclusion of GLONASS does not improve visibility in the critical areas, as expected based on Figure 8 and Table 4. Correspondingly, the LC GG/INS solution shows errors similar to the GPS-only case. Furthermore, the inclusion of the constraints also shows large disagreements with the reference during the long GNSS outages.

#### 5.1.3. Segment 3

**Figure 12.**
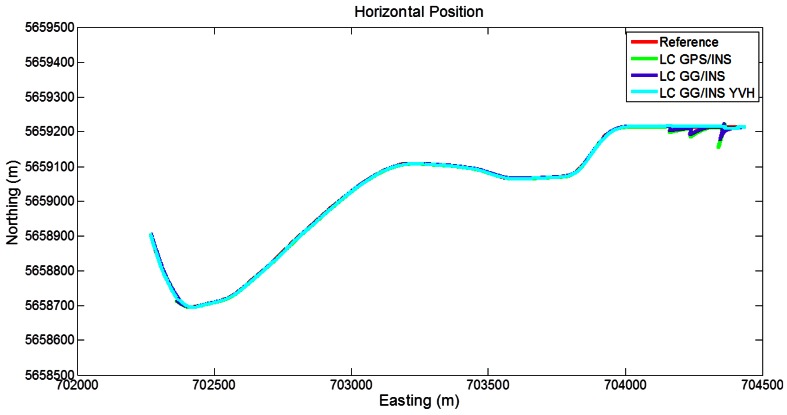
Trajectories obtained with the loose coupling approach (Segment 3).

In Figure 12 the trajectories obtained with the different LC approaches are shown. During the beginning of the segment, the GPS/INS shows some disagreements with the reference, due to the partial GPS outages. However, during the remaining part of the segment, with a good satellite visibility, the obtained trajectory is very close to the reference. Including the GLONASS measurements yields a reduction of the initial disagreements, as expected. With the addition of the GNSS-derived yaw aiding and the motion constraints on velocity and height, the obtained trajectory is close to the reference during the whole segment.

### 5.2. Tight Coupling Solutions

#### 5.2.1. Segment 1

**Figure 13.**
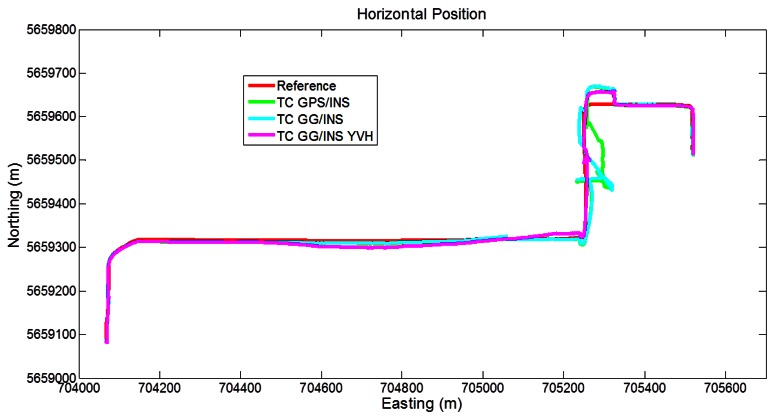
Trajectories obtained with the tight coupling approach (Segment 1).

In Figure 13 the trajectories obtained with the different TC approaches are shown. Compared with the LC results in Figure 10, all of the solutions show a significant improvement. This is consistent with previous studies and is attributed to the use of GNSS data when a few as one satellite is visible. In particular, the baseline configuration (TC GPS/INS) does not contain a large drift during the long GPS outage on the north/south portion of the trajectory. In this case, although including GLONASS observations still yields a better overall solution, the relative improvement is less than in the loose integration case. This is again attributed to the increased observability resulting from the tight integration. Finally, the trajectory obtained by also integrating the yaw aiding and the velocity and height pseudo-observations shows remarkable improvements relative to the other TC configuration especially during the above-defined critical zone.

The performance of different TC configurations, in terms of RMS and maximum position errors, is summarized in Table 6.

#### 5.2.2. Segment 2

**Figure 14.**
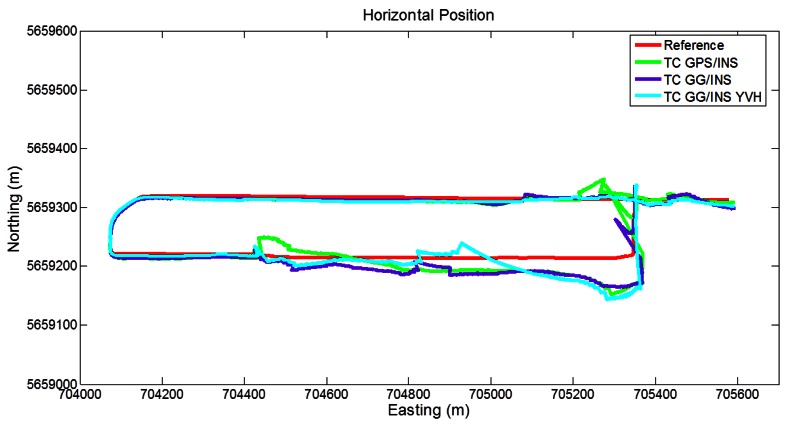
Trajectories obtained with the tight coupling approach (Segment 2).

The trajectories obtained with the different TC approaches for this scenario are shown in Figure 14. The GPS/INS solution does not present large errors during the GPS outages as in the corresponding LC case, owing to the ability to compute an integrated position in case of partial GNSS outages. A slightly better trajectory is obtained by including GLONASS measurements and the considered constraints.

#### 5.2.3. Segment 3

**Figure 15.**
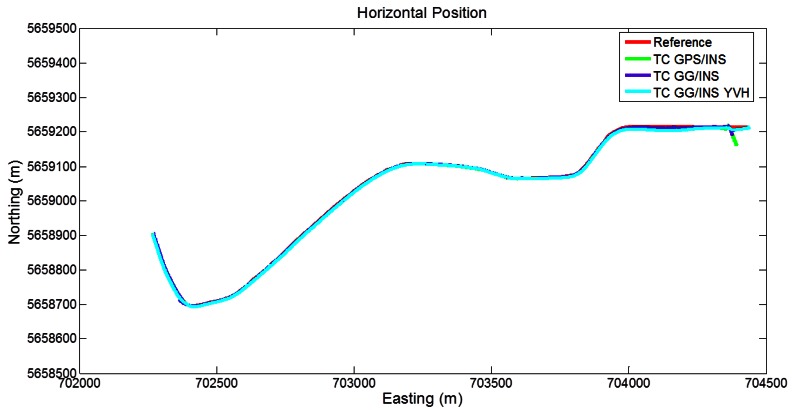
Trajectories obtained with the tight coupling approach (Segment 3).

The trajectories obtained with the different TC approaches for this scenario are presented in Figure 15. As expected, the results are very close to the reference due to the good GNSS coverage. The GPS/INS solution shows some disagreements during the GPS outages at the beginning of the segment. Again, including the GLONASS measurements yields a reduction of the above mentioned disagreements. Adding the GNSS-derived yaw aiding and the velocity and height motion constraints, the obtained trajectory is close to the reference during the whole segment.

### 5.3. Comparison of Loose and Tight Solutions

This section compares the loose and tight solutions for the different segments of the test.

#### 5.3.1. Segment 1

**Figure 16.**
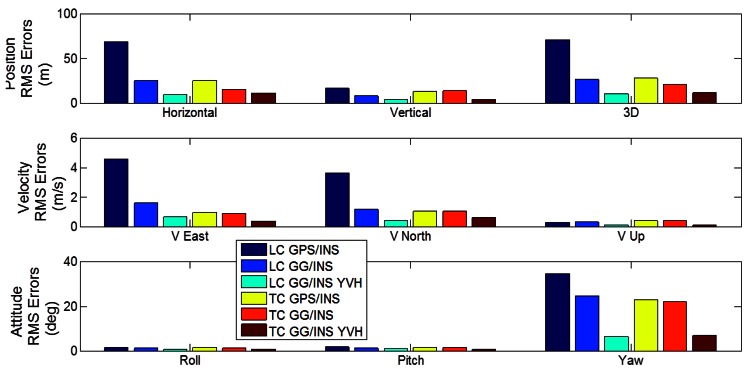
Comparison between LC and TC architectures in terms of position, velocity and attitude RMS errors (Segment 1).

To better perform a comparison among the analyzed configurations with LC and TC architectures, the RMS errors of position, velocity and attitude are presented in Figure 16. The main remarks deduced from this figure are:

- in the baseline GPS/INS integration, TC architecture provides significantly better horizontal solution and similar altitude result than LC, as expected and as shown in previous work;
- including GLONASS observations provides meaningful performance improvements for both LC and TC architectures in terms of position, velocity and azimuth estimation;
- in case of GPS/GLONASS without other aiding, the TC architecture provides only slightly better solution relative to the LC case;
- the loosely coupled GPS/GLONASS and tight coupled GPS-only configurations (no other aiding) provide very similar performance;
- including GNSS-derided azimuth aiding and velocity/height constraints produces significant improvements for both the LC and TC cases in terms of position, velocity and azimuth;
- the results obtained with using loose and tight integration for the GG/INS YVH configurations are very similar.

The practical consequences of the above considerations are two-fold. First, in this type of environment, the loosely coupled GPS/GLONASS configuration could replace the tightly coupled GPS-only configuration without performance degradation. Such an approach is simpler to implement thus saving development costs. Furthermore, the GNSS-only solution (*i.e.*, no inertial) is available in this case as well. Second, adding vehicle constraints is equally beneficial to the LC and TC cases, further blurring the benefits of the TC approach.

#### 5.3.2. Segment 2

**Figure 17.**
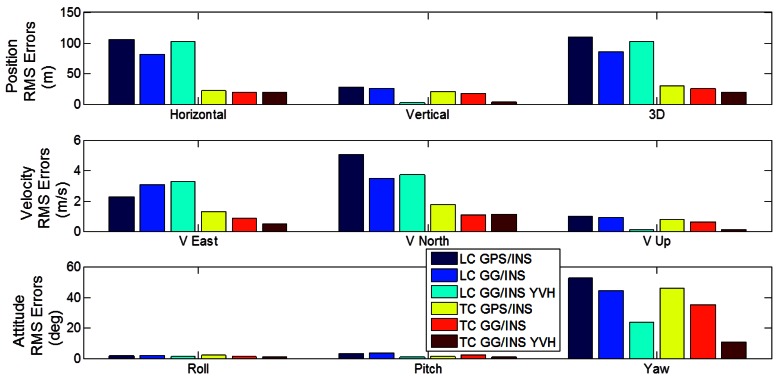
Comparison between LC and TC architectures in terms of position, velocity and attitude RMS errors (Segment 2).

The RMS errors of LC and TC architectures for segment 2 are presented in Figure 17. The main remarks deduced from this figure are:

- The LC architecture does not provide satisfying performance for each tested configuration (with GPS and GLONASS or with motion constraints), showing large errors during GNSS outages;
- The TC architecture shows better performance relative to LC architecture for each tested configuration;
- Including GLONASS observations provides slight performance improvements for TC architecture in terms of position, velocity and azimuth estimation;
- Including the GNSS-derived yaw aiding and velocity and height constraints in the TC GG/INS configuration improves the position, velocity and azimuth estimation.

From these results, unlike in segment 1, it is clear that a tight integration is still preferred since it offers significantly better performance overall.

#### 5.3.3. Segment 3

**Figure 18.**
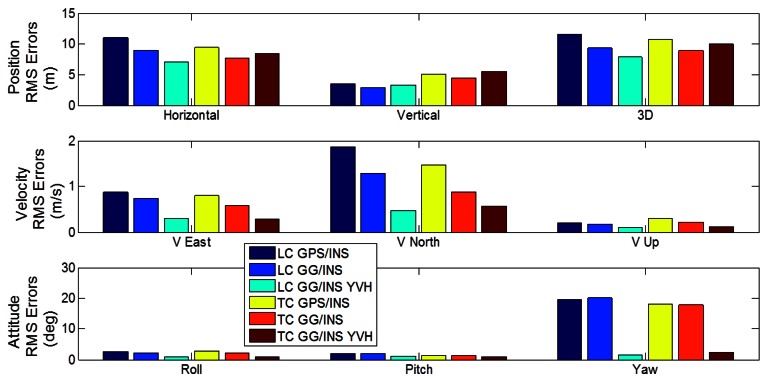
Comparison between LC and TC architectures in terms of position, velocity and attitude RMS errors (Segment 3).

The RMS errors of LC and TC architectures for this segment are shown in Figure 18. The main remarks deduced from this figure are:

- In this relatively benign scenario the position performance of all the considered configurations are very similar;
- Including GLONASS observations for both LC and TC architectures provides slight improvements in terms of position and velocity;
- Including GNSS-derived yaw aiding and velocity/height constraints yields the reduction of velocity and azimuth errors, but no benefits in position RMS errors.

As with segment 1, these findings suggest that using a GPS/GLONASS receiver in a LC system should yield results similar to the TC case, but with a simpler system and reduced development time.

To assess the overall performance of the considered configurations, the RMS errors for the three segments are shown in Table 7.

## 4. Conclusions

This work looks at the benefit of including GLONASS in integrated GPS/INS systems, especially in urban canyon environments. The effect of using different vehicle motion constraints was also considered. Data was collected in downtown Calgary and processed using various configurations. For the data analyzed herein, the main conclusions are as follows:

- In environments where satellite visibility is insufficient for standalone GNSS positioning approximately 50% of the time, the benefits of GLONASS are minimal in a loosely coupled implementation. However, in a tightly coupled implementation, GLONASS provides considerable improvements. In this respect, these results are similar to previous results obtained with GPS/INS systems.
- In environments when standalone GNSS positioning is possible 70% of time or more, inclusion of GLONASS in a loose integration provides similar performance to the GPS-only tight coupling system. This suggests that a simpler system is possible without sacrificing navigation performance simply by adding GLONASS measurements. In turn, this has direct benefits to system development time and cost.
- In benign environments where GNSS solutions are available most of the time, including GLONASS provides little benefit since the system is already dominated by the GNSS errors since free-inertial navigation is unnecessary.
- Including the GNSS-derived azimuth aiding and the velocity/height pseudo-observations produces significant performance improvements regardless of the integration strategy. Furthermore, results are quite similar between the loose and tight integrations in this case, thus further blurring the benefits of the tight integration approach.

Based on these results, inclusion of GLONASS observations does offer some significant advantages over GPS-only integrated systems. Although not quantified here, similar results would also be expected by including data from other GNSS as well (e.g., Galileo).

## Figures and Tables

**Table 1.** Summary of IMU characteristics for different grades of sensors (from [6,27]).

**Parameter**	**IMU Grade**		

	**Navigation**	**Tactical**	**MEMS**
**Accelerometers**			
In-Run Bias (mg)	0.025	1	2.5
Turn-On Bias (mg)	-	-	30
Scale Factor (PPM)	100	300	10,000
VRW (g/√Hz)	-	2.16e−06	370e−06

**Gyros**			
In-Run Bias (°/h)	0.0022	1	<1,040
Turn-On Bias (°/h)	-	-	5,400
Scale Factor (PPM)	5	150	10,000
ARW (°/h/√Hz)	6.92	7.5	226.8
Approx. Cost	>$90,000	>$20,000	<$2,000

**Table 2.** Crista IMU random noise spectral density and Gauss-Markov parameters.

**Accelerometers**					**Gyros**				

**Bias GM Parameters**		**Scale Factor GM Parameters**		**Noise**	**Bias GM Parameters**		**Scale Factor GM Parameters**		**Noise**
*σ*(*m*/*s*^2^)	*τ*(*s*)	*σ*(*PPM*)	*τ*(*s*)	$g/\sqrt{Hz}$	*σ*(deg/*s*)	*τ*(*s*)	*σ*(*PPM*)	*τ*(*s*)	$(\deg/s)/\sqrt{Hz}$
0.0077	270	10,000	18,000	300e−6	192	350	10,000	18,000	220

**Table 3.** Reference solution accuracy.

**Information**	**Accuracy**
Position	dm level
Velocity	cm/s level
Attitude	<1 deg

**Table 4.** GNSS availability and outage duration.

**Segment**	**GNSS Constellation**	**Solution Availability**	**Longest Solution Outage**
1	GPS	73%	60 s
	GPS/GLONASS	81%	30 s
2	GPS	53%	60 s
	GPS/GLONASS	55%	60 s
3	GPS	81%	10 s
	GPS/GLONASS	86%	8 s

**Table 5.** Position error obtained with LC configurations.

**Configuration**	**Position Error (m)**					

	***RMS***			***Maximum***		

	***East***	***North***	***Up***	***East***	***North***	***Up***
LC GPS/INS	48.4	48.7	16.3	347.0	341.2	48.0
LC GG/INS	20.2	15.0	8.0	109.6	51.2	34.4
LC GG/INS Y	15.0	12.9	7.6	80.1	48.2	33.1
LC GG/INS YV	8.1	6.6	3.8	41.9	40.0	8.8
LC GG/INS YVH	7.9	5.6	3.9	33.9	20.5	6.3

**Table 6.** Position error obtained with TC configurations.

**Configuration**	**Position Error (m)**					

	***RMS***			***Maximum***		

	***East***	***North***	***Up***	***East***	***North***	***Up***
TC GPS/INS	14.1	20.4	13.3	61.4	70.1	55.7
TC GG/INS	8.2	12.9	13.0	61.1	69.7	46.4
TC GG/INS YVH	4.8	9.5	4.0	18.4	30.5	6.4

**Table 7.** Performance comparison between LC and TC configurations for the three segments.

**Configuration**	***RMS***								

	**Position Error (m)**			**Velocity Error (m/s)**			**Attitude Error (deg)**		

	***East***	***North***	***Up***	***East***	***North***	***Up***	***Roll***	***Pitch***	***Yaw***
LC GPS/INS	37.8	72.2	20.8	3.30	4.06	0.68	1.9	2.5	41.2
LC GG/INS	36.6	41.0	17.3	2.24	2.41	0.63	1.8	2.5	33.8
LC GG/INS YVH	37.1	42.5	3.2	2.14	2.41	0.10	1.1	1.1	16.0
TC GPS/INS	11.8	18.4	15.8	1.08	1.44	0.57	2.2	1.5	33.9
TC GG/INS	8.6	13.8	14.1	0.84	1.03	0.47	1.6	1.8	27.8
TC GG/INS YVH	6.2	13.5	4.1	0.41	0.87	0.11	1.0	1.0	8.4
